# Sequential occurrence of *BCR::ABL1*-negative MPN and CML and vice versa: results from a real world cohort

**DOI:** 10.1007/s12185-025-04046-5

**Published:** 2025-09-04

**Authors:** Katrin Schweneker, Miriam Lenk, Wolfgang Kern, Claudia Haferlach, Manja Meggendorfer, Christian Pohlkamp, Torsten Haferlach

**Affiliations:** MLL Munich Leukemia Laboratory, Max-Lebsche-Platz 31, 81377 Munich, Germany

**Keywords:** CML, *BCR*::*ABL1* negative-MPN, Sequential myeloproliferative disorders, *JAK2*, Myeloid malignancies

## Abstract

Chronic myeloid leukemia (CML) and *BCR*::*ABL1*-negative MPN were thought to be mutually exclusive, but synchronous and sequential cases have been reported. We screened 35,001 patients for *BCR*::*ABL1* fusion or *JAK2*, *CALR*, or *MPL* mutations to investigate the sequential development of CML and *BCR*::*ABL1* negative-MPNs. We discovered that 5.6% had primary CML followed by *BCR*::*ABL1*-negative MPN, and 5.8% had the reverse sequence. Notably, we identified higher *JAK2* variant allele frequencies (VAFs) in patients developing secondary CML. Previous MPN did not compromise the effectiveness of tyrosine kinase inhibitors (TKI) in treating secondary CML. The emergence of secondary MPN appeared to be unrelated to *JAK2* VAF progression or *BCR*::*ABL1* transcript levels. Our research indicates that newly detected leukocytosis or thrombocytosis should prompt consideration of secondary MPN. It also showed that secondary CML had no negative impact on response to therapy when patients were treated according to CML guidelines.

**Supplementary Information:**

The online version contains supplementary material available at 10.1007/s12185-025-04046-5.

## Introduction

Myeloproliferative neoplasms (MPN) are a heterogeneous group of hematological disorders defined by clonal proliferation of blood cells. *BCR::ABL1* fusions define CML, whereas mutations in the genes *JAK2*, *CALR,* or *MPL* are associated with most of the *BCR::ABL1*-negative MPN [1–4]. While CML and *BCR::ABL1*-negative MPN have been considered to be mutually exclusive [5], the WHO classification requires the exclusion of the *BCR::ABL1* fusion in all cases of suspected MPN that cannot be clinically classified as Polycythemia vera [6]. In recent years, many cases have been reported of rare events of synchronous or sequential occurrence of CML and *BCR::ABL1*-negative MPN clones [7]. The relationship between synchronously diagnosed CML and *BCR::ABL1*-negative MPN was investigated in a report of 23 patients that we published in 2016 [8]. In the present publication, we analyzed the relationship between two MPN clones in patients with sequential diagnosis of CML and *BCR::ABL1*-negative MPN and vice versa in a retrospective study.

## Material and methods

35,001 patients with *BCR::ABL1*-negative MPN or CML were diagnosed at our institution between August 2005 and January 2023. Of these, 27,896 patients showed a *JAK2*, *CALR,* and/or *MPL* mutation, indicating the diagnosis of a *BCR::ABL1*-negative MPN. 7,105 patients had a *BCR::ABL1* fusion, confirming the diagnosis of CML. During follow-up, changes in blood counts suggested a secondary *BCR::ABL1*-negative MPN or CML. Progression of CML was ruled out based on the detection of a stable or decreasing *BCR::ABL1* ratio. Therefore, repeat testing for *JAK2*, *CALR,* and *MPL* mutations was initiated in 337 patients with CML and repeat testing for the *BCR::ABL1* fusion was performed in 953 patients with *BCR::ABL1*-negative MPN, respectively. Among patients with CML, 318 patients were negative for *JAK2*, *CALR,* or *MPL* mutation (CML), whereas 19 patients (5.6%) with CML were positive for *JAK2*, *CALR,* or *MPL* mutation and had acquired a secondary *BCR::ABL1*-negative MPN (CML + MPN). In the group of patients with *BCR::ABL1*-negative MPN, 898 patients were negative for the *BCR::ABL1* fusion (MPN). Only 55 patients (5.8%) were positive for the *BCR::ABL1* fusion and had a secondary CML (MPN + CML, Supplemental Fig. 1). Patients published by Martin-Cabrera P. et al*.* were excluded [8]. Additionally, cases of CML that were not tested for MPN drivers and cases of MPN that were not tested for *BCR::ABL1* at the time of initial diagnosis were excluded.

All patients had given written informed consent for the use of genetic and clinical data in accordance with the Declaration of Helsinki. The study was approved by our internal institutional review board. Samples were analyzed by either Sanger sequencing or next-generation sequencing (NGS) as previously described [9]. Disease progression was assessed by reverse transcription quantitative real-time PCR (q-PCR). Statistical analysis was performed using GraphPadPrism and Mann–Whitney U Test.

## Results

**Fig. 1:**
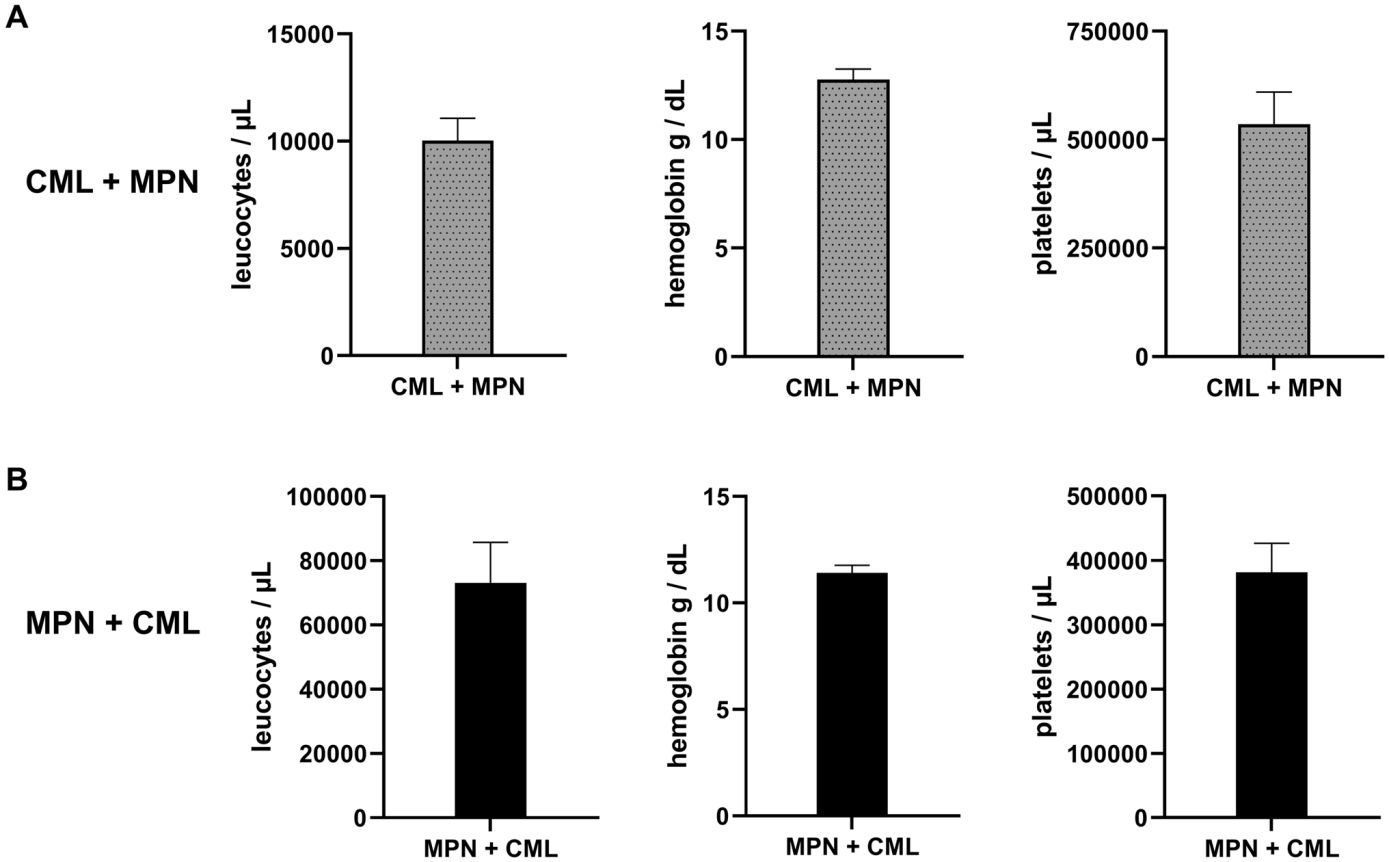
Analysis of changes in peripheral blood count that prompted testing for secondary *BCR::ABL1*-negative MPN or CML. **A**, 13 patients with CML + *BCR::ABL1*-negative MPN showed mainly thrombocytosis, while leukocytes were only slightly elevated on average and hemoglobin was in the normal range. **B**, 43 patients with *BCR::ABL1*-negative MPN + CML showed mainly leukocytosis, while hemoglobin was slightly decreased on average and platelets were in the upper normal range on average.

### Changes of peripheral blood count that prompted testing for secondary *BCR::ABL1*-negative MPN or CML

We analyzed leukocyte and platelet counts as well as hemoglobin at the time of suspected secondary MPN. Of the patients in the CML + MPN group, blood counts were available for 13 out of 19 patients. These exhibited mainly thrombocytosis with a mean of 535,923 platelets/µl (range 270.000–1.108.000/µl), leukocytes were only slightly elevated with a mean of 10,028 leukocytes/µl (range 5.400–26.200/µl) whereas the mean hemoglobin was 13 g/dl (range 9.9–16.2 g/dl, Fig. 1A). In contrast, 43 out of 55 with peripheral blood counts and differentials were available in the group of patients with MPN + CML and these had a marked leukocytosis of mean 73,144/µl (range 3800–420,270/µl), whereas the mean hemoglobin was slightly reduced with 11.0 g/dl (range 7.2–15.1 g/dl) and the mean platelet count was 381,333 platelets/µl (range 17,000–1,126,000/µl; Fig. 1B).

#### Gender and age and impact of time between first and secondary diagnosed MPN on development of secondary *BCR::ABL1-*negative MPN or CML

**Fig. 2:**
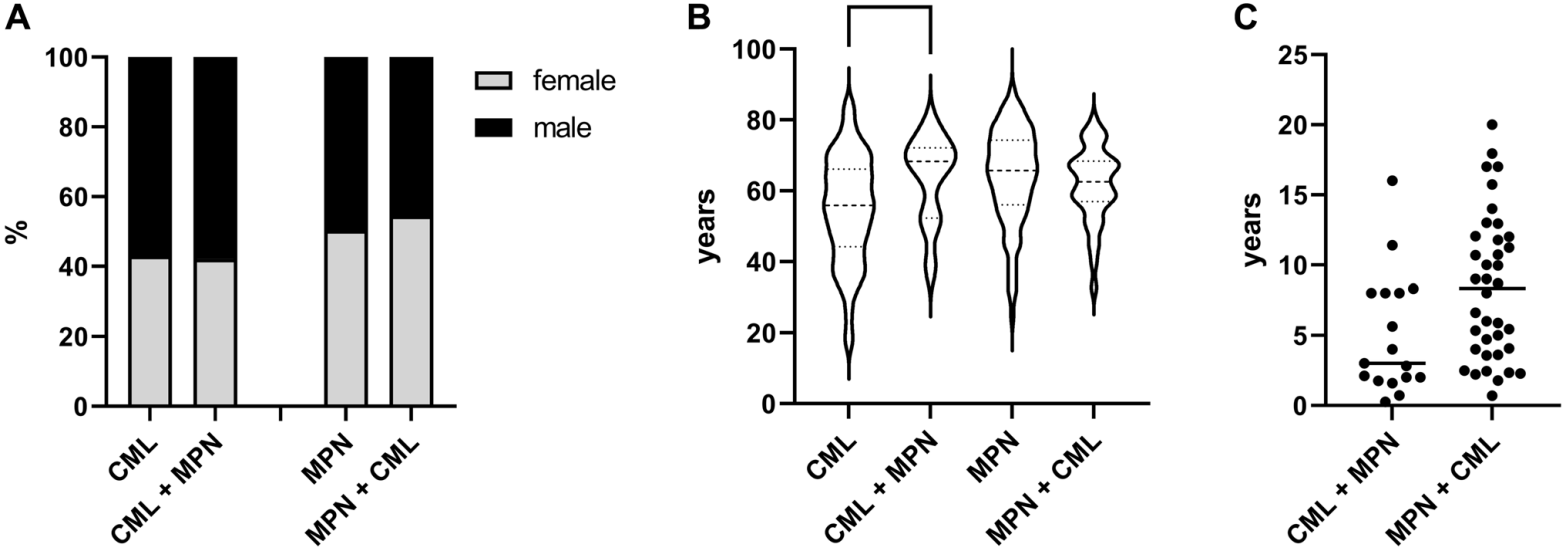
Analysis of distribution of sex and age and impact of time between first and secondary diagnosed MPN on development of secondary *BCR::ABL1*-negative MPN or CML. **A**, The distribution of sex was similar in the patients with CML + MPN and MPN + CML compared to their control groups. **B**, There were no significant differences in the age at the time point of first diagnosed CML or *BCR::ABL1*-negative MPN compared to their control groups (ns not significant). **C**, Time between first and secondary diagnosed CML or *BCR::ABL1*-negative MPN had a wide range without a critical time point for frequent diagnoses of secondary *BCR::ABL1*-negative MPN or CML

To address the question of whether there are risk factors for the development of secondary *BCR::ABL1*-negative MPN or CML, we examined the gender and age distribution of the patients at the time of primarily diagnosed *BCR::ABL1*-negative MPN or CML. Among patients with CML + MPN, 42.1% were female and 57.9% were male, a similar distribution compared to the control group of CML patients (43.0% female and 57.0% male). Likewise, in the group of patients with MPN + CML, 54.5% were female and 45.5% male, which was comparable to the control group of patients with MPN (50.37% female and 49.63% male; Fig. 2A). Age at primary diagnosis was not significantly different between patients with *BCR::ABL1*-negative MPN or CML and patients with CML + MPN and MPN + CML, respectively. Patients with CML + MPN had a median age of 66.72 years (range 38.9–78.3 years) at CML diagnosis, which was not significantly different from patients with CML who had a median age of 58.4 years (range 15.8–85.9 years) at CML diagnosis. Similarly, patients with MPN + CML and patients with MPN did not differ in median age at diagnosis of MPN (61.6 years vs. 64.2 years, range 14.3–82.5 years and 22.5–72.8 years, respectively, Fig. 2B).

A further risk factor for the development of secondary *BCR::ABL1*-negative MPN or CML could be the time after diagnosis. To address this issue, we analyzed the time between the first and second diagnosis of MPN. The mean time between diagnosis of CML and *BCR::ABL1*-negative MPN was 4.78 years with a range of 0.27–16.0 years. In the case of a known *BCR::ABL1*-negative MPN, the mean time to diagnosis of CML was 8.4 years (range 0.7–20.0 years). These results suggest that there is no specific time period for the appearance of secondary *BCR::ABL1*-negative MPN or CML in either group (Fig. 2C).

#### Influence of driver mutation, *JAK2* mutational burden, and CML remission status on development of secondary CML or *BCR::ABL1-*negative MPN

**Fig. 3:**
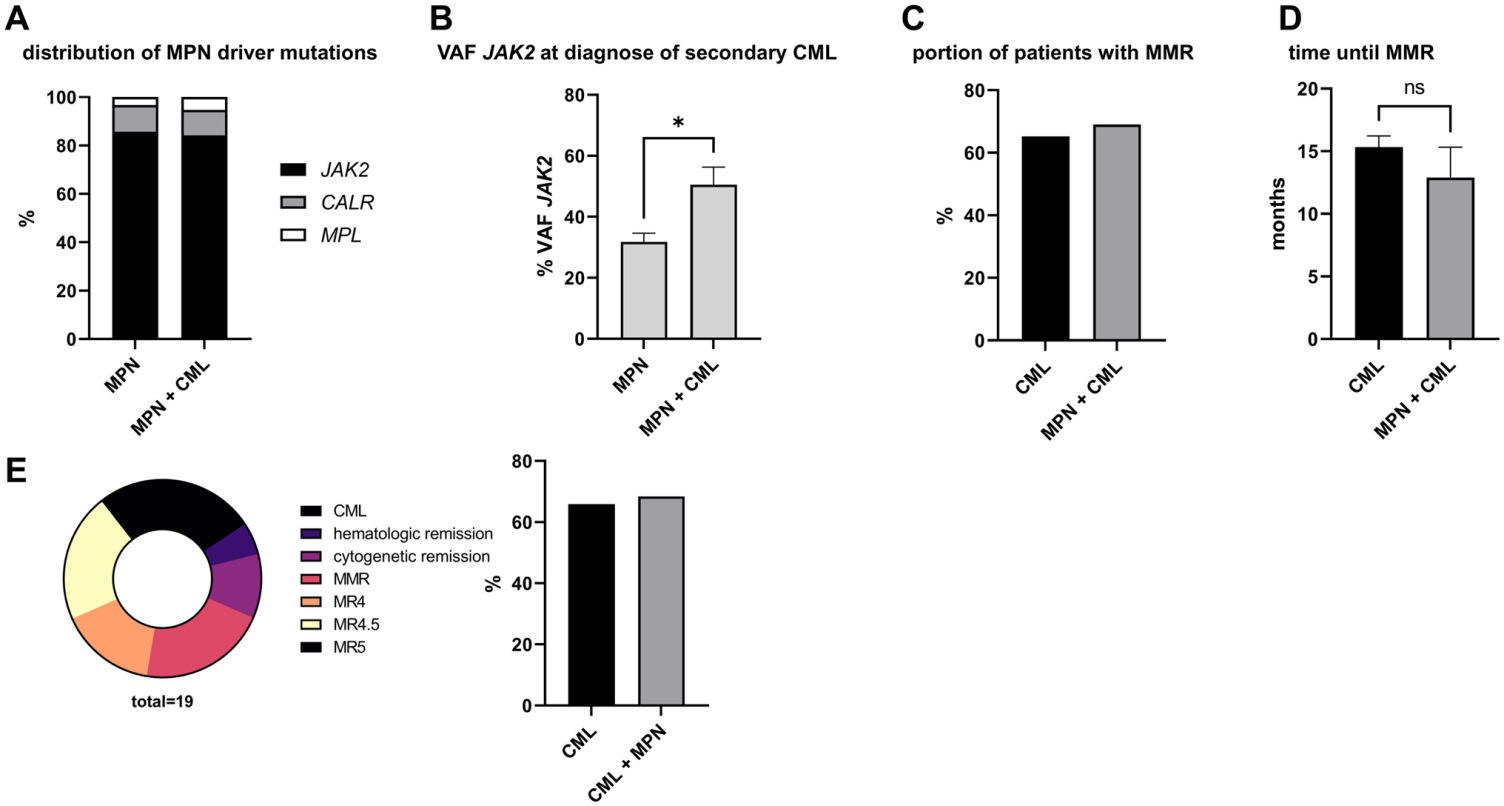
Influence of kind of driver mutation, mutational burden of *JAK2,* and remission status of CML on development of secondary CML or *BCR::ABL1*-negative MPN. **A**, The distribution of driver mutations was comparable to the control group. **B**, Patients with *BCR::ABL*-negative MPN and secondary CML had a significantly higher VAF of *JAK2* at the time point of diagnosed CML compared to the control group. **C**, The portion of patients with MPN + CML that reached MMR under treatment with TKI was similar to the control group. **D**, In the group of patients with MPN + CML, the time until reaching MMR under treatment with TKI was comparable to the control group. **E**, The portion of patients with MMR or better in the group of patients with CML + MPN when *BCR::ABL1*-negative MPN was diagnosed was similar to the control group.

Previous publications have shown that the type of driver mutation and a higher VAF of *JAK2*, *CALR,* or *MPL* have an impact on the course of *BCR::ABL1*-negative MPN [10–12]. Therefore, we investigated the distribution of mutations in *JAK2*, *CALR,* and *MPL* in patients with MPN + CML and the mutational burden of *JAK2* at the time of secondary diagnosis of CML. The distribution of mutations in *JAK2*, *CALR,* or *MPL* was comparable between patients with MPN + CML and patients with MPN. Most patients had a mutation in *JAK2* (MPN + CML: 80.0%, MPN: 86.5%), followed by mutations in *CALR* (MPN + CML: 14.5%, MPN: 11.1%) and *MPL* (MPN + CML: 5.5%, MPN: 3.3%). This distribution is consistent with previously published data [13] and indicates that the type of driver mutation is not a risk factor for the development of secondary CML (Fig. 3A). Furthermore, we analyzed details regarding the treatment of *BCR::ABL1*-negative MPN at the time of secondary CML diagnosis (Supplemental Fig. 2). Data were available for 36 out of 55 patients, all of whom were treated with therapies recommended by the guidelines if therapy was indicated. Due to the small cohort of patients with MPN + CML, we could only analyze the VAF of *JAK2* mutation at the time of diagnosis of secondary CML. In patients with MPN + CML, the mean VAF of *JAK2* mutation was significantly higher than in patients with MPN (52.6% vs. 29%, *p* = 0.0307, Fig. 3B). These findings suggest that a higher VAF of the *JAK2* mutation could potentially promote the development of secondary CML.

In CML, response to treatment with tyrosine kinase inhibitors (TKI), as measured by *BCR::ABL1* transcript levels is known to be the most important prognostic factor [14]. We wondered whether patients with MPN + CML had a worse response to TKI therapy or needed more time to achieve MMR than the control group. After a mean of 12.9 months (range 3–49 months), 69.0% of the patients with MPN + CML achieved MMR or better, which is similar to patients with CML. In this group, 65.2% patients reached MMR or better in a mean time of 15.1 months (range 12.1–166 months, Fig. 3C and D).

Patients with CML who do not achieve MMR on TKI treatment have a worse outcome [15]. Comparable to the higher VAF of *JAK2* in patients with *BCR::ABL1*-negative MPN, the higher *BCR::ABL1* transcript level might be a risk factor for secondary *BCR::ABL1*-negative MPN in patients with CML. Therefore, we investigated the remission status of CML at the time of secondary diagnosis of *BCR::ABL1*-negative MPN. In total, 68.4% of patients had an MMR or better, which is similar to the control group CML with 65.9% (Fig. 3E).

We wondered if patients with MPN + CML or CML + MPN are more likely to develop *BCR::ABL1* mutations. Mutation analysis was performed in 16 cases from our cohort of 74 patients (10 with MPN + CML, 6 with CML + MPN). Mutations were found in 1 case of MPN + CML (E255K and G250E mutations) and in 1 case of CML + MPN (p.Q252H mutation). Due to the small number of patients, we cannot draw profound conclusions from this data.

#### Dependency of the CML and the *JAK2* clones on each other during course of disease and evaluation of treatment of patients with CML + MPN and MPN + CML

**Fig. 4:**
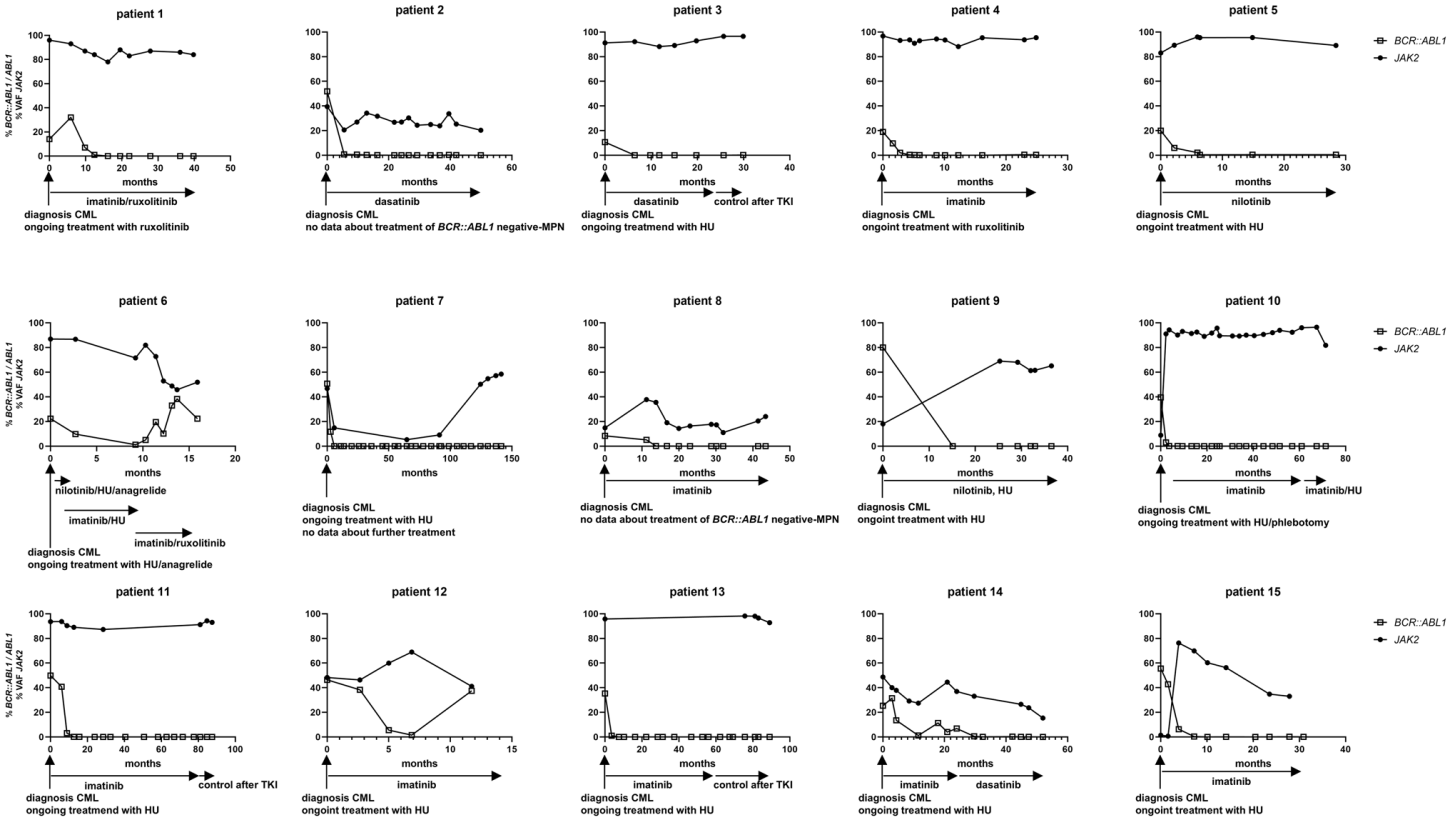
Study of dependency of the CML- and the *JAK2*-clone during course of disease. HU hydroxyurea. Most patients with MPN + CML did not show a connection between the course of *BCR::ABL1* transcript level and VAF *JAK2* in the follow-up

The association between the two different MPN clones in cases of coexisting CML and *BCR::ABL1*-negative MPN is controversially discussed in the literature [8, 16]. To investigate the connection between the *BCR::ABL1* fusion clone and the *JAK2*-mutated clone, we analyzed the mutational burden of *JAK2* at diagnosis of *BCR::ABL1-*negative MPN and at diagnosis of CML in 7 patients. At the time of diagnosis of CML, 3 patients (pat. no 2, 3, 7) showed a stable VAF of *JAK2* mutation, 2 patients (pat. no 1, 4) showed a higher VAF of *JAK2* mutation*,* and 2 patients (pat. no 5, 6) had a lower VAF of *JAK2* mutation compared to the VAF at time of diagnosis of *JAK2*-mutated MPN (Table 1). Furthermore, we analyzed the course of VAF of *JAK2* mutations during CML therapy with TKI in 15 patients with MPN + CML. Most patients (*n* = 10, 67%) showed an independent course of the VAF of *JAK2* mutations and *BCR::ABL1* transcript levels (pat. no 1–5, 9–13, Fig. 4). While *BCR::ABL1* transcript levels decreased with TKI treatment, the VAF of *JAK2* was not affected by this therapy or increased during the course of treatment. In these cases, a clear influence of the *BCR::ABL1*-negative MPN specific therapy (ruxolitinib or hydroxyurea) on *JAK2* VAF was not observed. Due to the small number of patients, this observation is limited to patients 1, 9, and 10, who were treated with TKI and ruxolitinib or hydroxyurea. In all other cases (pat. no 6–8, 14, 15), *JAK2* VAF decreased after TKI therapy was initiated. Patient 6 was the only one who received treatment of *BCR::ABL1*-negative MPN with hydroxyurea, followed by ruxolitinib. In all other cases, decreasing *JAK2* levels were not induced by *BCR::ABL1*-negative MPN specific therapy. These data support the theory that the two different MPN clones can be independent of each other, but there are exceptions with a possible dependency of the mutational burden of *JAK2* mutation and *BCR::ABL1* transcript level.

**Table 1:** Influence of mutational burden of *JAK2* on development of secondary CML.

patient no	time in years between diagnosis of MPN and secondary CML	*% BCR::ABL1* ratio	% VAF *JAK2*
1	3.13	0	31
		14.03	56.61
2	5.1	0	19
		49.78	22.5
3	5.4	0	82.74
		19.98	83
4	5.5	0	49.1
		22.29	86.87
5	2.2	0	90.41
		39.52	8.9
6	4.1	0	86.39
		72.7	8.0
7	1.8	0	23.10
		56.25	25

At the time of diagnosis of CML, 3 patients (pat. no 2, 3, 7) showed a stable VAF of *JAK2* mutation, 2 patients (pat. no 1, 4) showed a higher VAF of *JAK2* mutation, and 2 patients (pat. no 5, 6) had a lower VAF of *JAK2* mutation compared to the VAF at time of diagnosis of *JAK2*-mutated MPN

Furthermore, we analyzed data on the treatment of patients with MPN + CML or CML + MPN following a diagnosis of secondary CML or *BCR::ABL1*-negative MPN (Supplemental Fig. 3). During follow-up, data from 56 patients (14 patients with CML + MPN and 42 patients with MPN + CML) were available, and all received treatment according to the CML and MPN guidelines if therapy was indicated.

## Discussion

In the literature, the sequential occurrence of *BCR::ABL1*-negative MPN and CML or vice versa is mainly described in case reports [7]. There is only one larger study published by Hochman et al*.* analyzing data from the MPN registry of the Sidney Kimmel Comprehensive Cancer Center (The Johns Hopkins University School of Medicine, Baltimore, USA). The incidence of secondary CML in case of an existing *BCR::ABL1*-negative MPN has been reported to be 0.6% [17]. In our study, we did not report the incidence because we cannot exclude the possibility that there are patients with secondary CML or *BCR::ABL1*-negative MPN in our cohort who were not tested in our laboratory during follow-up. Therefore, we restricted our analysis to the group of patients who were tested for the driver mutations or the *BCR::ABL1* fusion during the course of their disease. In this restricted analysis, the incidence of MPN + CML was 5.8%, confirming the data of Hochman et al*.* and showing that the risk for CML in patients with existing *BCR::ABL1*-negative MPN is much higher than the risk for CML in the general population [18]. In patients with CML + MPN, a study of 314 CML patients showed an incidence of 2.55% for the concomitant occurrence of a *JAK2* mutation [19]. This incidence is lower than in our study because testing for driver mutations was initiated regardless of disease progression. Our data suggest that more CML patients with secondary *BCR::ABL1*-negative MPN are diagnosed when testing for the *JAK2*, *CALR*, and *MPL* mutation is prompted by emerging changes in the blood count.

As previously published [17, 20], testing for *BCR::ABL1* fusion in patients with known *BCR::ABL1*-negative MPN was prompted by new-onset leukocytosis, whereas testing for driver mutations in patients with known CML was prompted by new-onset thrombocytosis. Our data support that physicians should be alerted to secondary CML or *BCR::ABL1*-negative MPN in case of new blood count abnormalities suggestive of MPN.

To our knowledge, our study is the first one to analyze the influence of gender and age on the sequential occurrence of secondary CML or *BCR::ABL1*-negative MPN. Our data did not show an impact of gender or age in this process further studies are needed to confirm these results. Hochman et al*.* published that the development of CML occurred no less than 10 and up to 36 years after the diagnosis of *BCR::ABL1*-negative MPN [17], whereas in our study, 0.7–20.0 years elapsed between the diagnoses of both MPNs. Since *BCR::ABL1* testing was prompted by a suspected clinical diagnosis of CML rather than being performed routinely during the course of *BCR::ABL1*-negative MPN, the exact onset of secondary CML cannot be determined and may have occurred earlier than molecularly diagnosed. Consequently, the precise timing of a detectable *BCR::ABL1* fusion, and thus the true onset of CML, remains uncertain in our cohort. This limitation may lead to an underestimation of the latency period between the initial diagnosis of *BCR::ABL1*-negative MPN and the emergence of secondary CML. Consequently, caution is warranted in interpreting the reported duration as a direct reflection of disease biology or progression dynamics. Future prospective studies with molecular monitoring may help to more accurately capture the timing of clonal evolution in such cases. In contrast to our study, Hochman et al*.* analyzed 4 patients with MPN + CML out of a cohort of 630 patients with *BCR::ABL1*-negative MPN, whereas we studied 55 patients with MPN + CML. Furthermore, follow-up was done with an average observation time of 4 years post-enrollment in the single-center registry and 9 years after MPN diagnosis. Patients with an earlier diagnosis of secondary CML may have been lost to follow-up. In patients with CML + MPN, the time between first and second diagnosis of MPN ranged from 0.27 to 16.0 years, which is comparable to the patients with MPN + CML in our study.

There is much debate in the literature about the relationship between two MPNs diagnosed at different times [8, 21]. Furthermore, there are publications showing that a higher VAF of *JAK2* mutation (> 50%) is associated with increased transformation to myelofibrosis as a sign of higher disease activity [22–24]. This is in line with our observation that a higher VAF of *JAK2* mutations results more often in secondary CML, implying that a higher activity of a *JAK2*-mutated MPN may be a driver for secondary CML and that the CML clone depends on the *JAK2*-mutated clone. In contrast, the response to treatment of CML was not negatively influenced by the higher mutational burden of *JAK2*-mutated MPN. Furthermore, the occurrence of secondary *BCR::ABL1*-negative MPN in patients with CML was not dependent on the level of *BCR::ABL1* transcript, implying that the activity of CML does not influence the development of secondary *BCR::ABL1*-negative MPN. Finally, we found no clear correlation between the mutational burden of the *JAK2* mutation and the *BCR::ABL1* transcript levels at the time of CML diagnosis and during treatment of CML with TKI. These data suggest that in most cases, both MPN clones are independent of each other, but there are exceptions where both MPN clones compete with each other.

## Conclusions

Our study is the first one to analyze the sequential occurrence of CML and *BCR::ABL1*-negative MPN and vice versa in a larger cohort. Our data do not show a clear relationship between two different sequentially developed MPN clones, but patients with *BCR::ABL1*-negative MPN and a higher VAF of *JAK2* mutation more often develop secondary CML. The response to treatment of CML was not negatively influenced by the higher mutational burden of *JAK2* in these patients. Finally, physicians should be alerted to the possibility of secondary CML or *BCR::ABL1*-negative MPN in patients with known MPN when there are new changes in the blood counts. Further studies are needed to better understand the relationship between these related myeloid diseases and to make recommendations for their therapeutic management.

## Supplementary Information

Below is the link to the electronic supplementary material.

Supplementary file1 (PPTX 47 KB)

## Data Availability

For original data, please contact corresponding author(katrin.schweneker@mll.com).
