# Supplementary material for: Sequential occurrence of BCR::ABL1-negative MPN and CML and vice versa: results from a real world cohort
Source: Int J Hematol. 2025 Sep 4;122(6):835–42. doi: 10.1007/s12185-025-04046-5 (PMC12638402; doi:10.1007/s12185-025-04046-5)
Supplement: Supplementary file 1 — Supplementary file1 (PPTX 47 KB) [file 12185_2025_4046_MOESM1_ESM.pptx]

## Slide 1
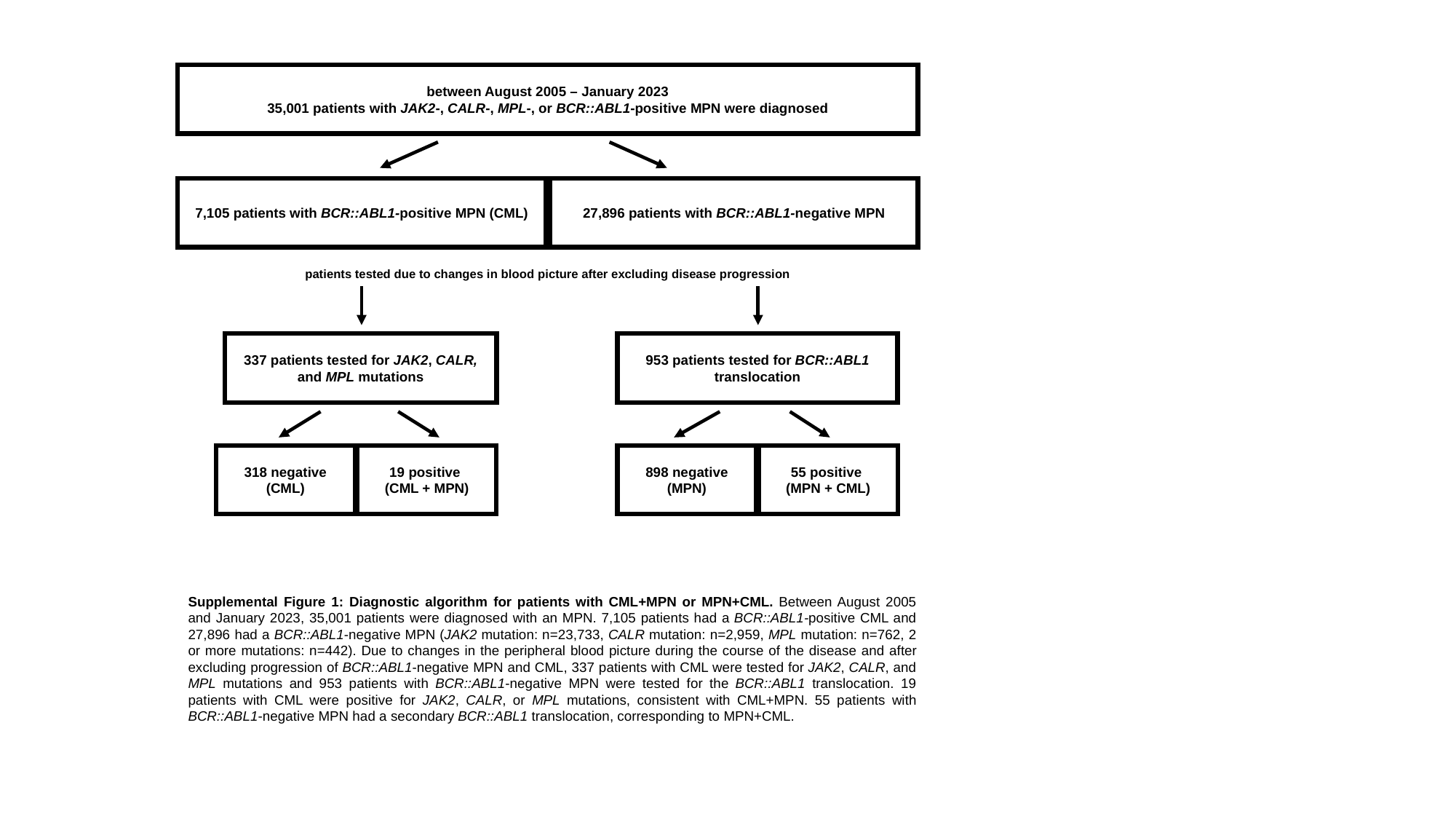

between August 2005 – January 2023
35,001 patients with JAK2-, CALR-, MPL-, or BCR::ABL1-positive MPN were diagnosed
7,105 patients with BCR::ABL1-positive MPN (CML)
27,896 patients with BCR::ABL1-negative MPN
patients tested due to changes in blood picture after excluding disease progression
953 patients tested for BCR::ABL1 translocation
337 patients tested for JAK2, CALR, and MPL mutations
318 negative (CML)
19 positive
(CML + MPN)
898 negative (MPN)
55 positive
(MPN + CML)
Supplemental Figure 1: Diagnostic algorithm for patients with CML+MPN or MPN+CML. Between August 2005 and January 2023, 35,001 patients were diagnosed with an MPN. 7,105 patients had a BCR::ABL1-positive CML and 27,896 had a BCR::ABL1-negative MPN (JAK2 mutation: n=23,733, CALR mutation: n=2,959, MPL mutation: n=762, 2 or more mutations: n=442). Due to changes in the peripheral blood picture during the course of the disease and after excluding progression of BCR::ABL1-negative MPN and CML, 337 patients with CML were tested for JAK2, CALR, and MPL mutations and 953 patients with BCR::ABL1-negative MPN were tested for the BCR::ABL1 translocation. 19 patients with CML were positive for JAK2, CALR, or MPL mutations, consistent with CML+MPN. 55 patients with BCR::ABL1-negative MPN had a secondary BCR::ABL1 translocation, corresponding to MPN+CML.

## Slide 2
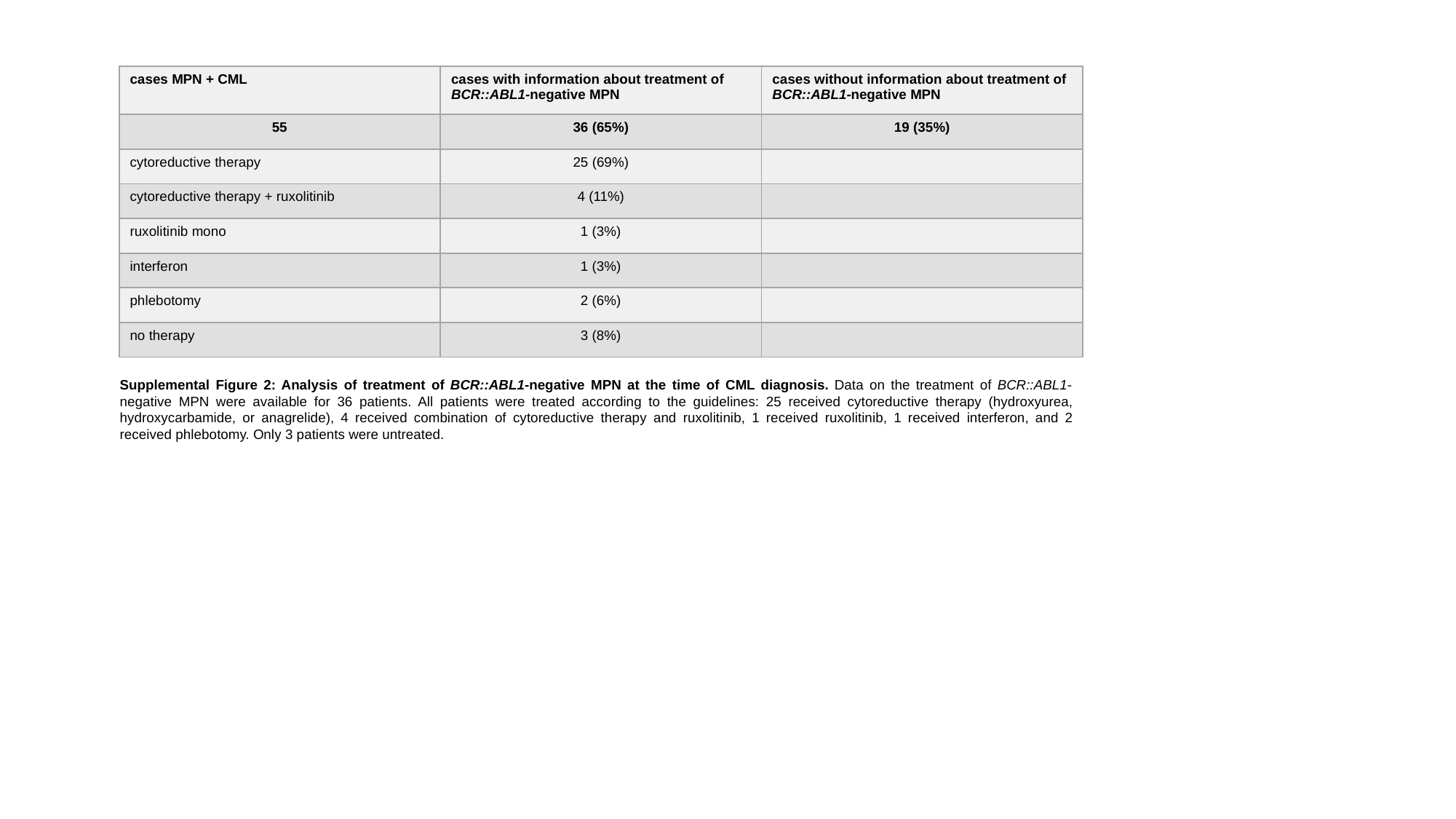

| cases MPN + CML | cases with information about treatment of BCR::ABL1-negative MPN | cases without information about treatment of BCR::ABL1-negative MPN |
| --- | --- | --- |
| 55 | 36 (65%) | 19 (35%) |
| cytoreductive therapy | 25 (69%) | |
| cytoreductive therapy + ruxolitinib | 4 (11%) | |
| ruxolitinib mono | 1 (3%) | |
| interferon | 1 (3%) | |
| phlebotomy | 2 (6%) | |
| no therapy | 3 (8%) | |
Supplemental Figure 2: Analysis of treatment of BCR::ABL1-negative MPN at the time of CML diagnosis. Data on the treatment of BCR::ABL1-negative MPN were available for 36 patients. All patients were treated according to the guidelines: 25 received cytoreductive therapy (hydroxyurea, hydroxycarbamide, or anagrelide), 4 received combination of cytoreductive therapy and ruxolitinib, 1 received ruxolitinib, 1 received interferon, and 2 received phlebotomy. Only 3 patients were untreated.

## Slide 3
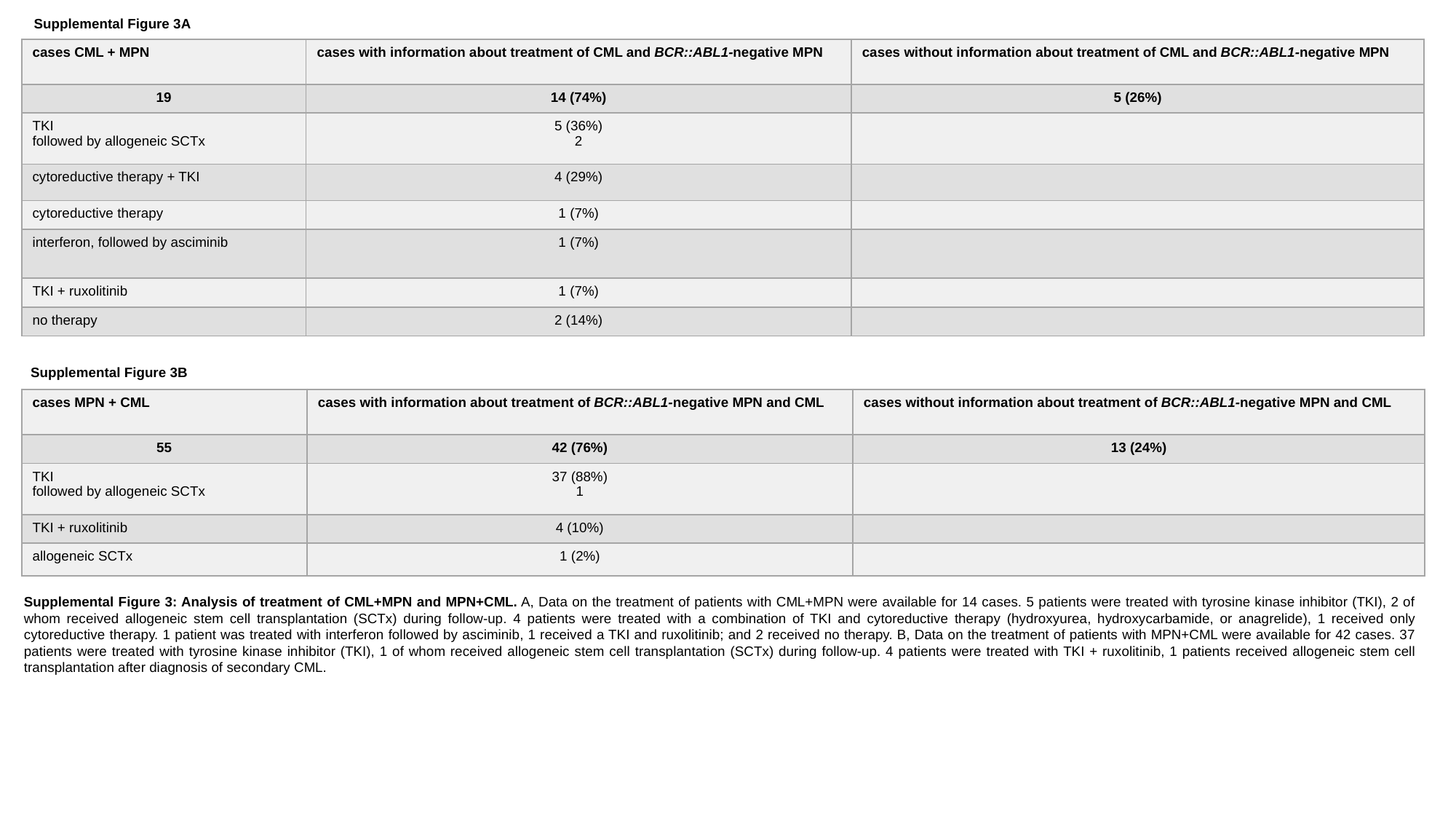

Supplemental Figure 3A
| cases CML + MPN | cases with information about treatment of CML and BCR::ABL1-negative MPN | cases without information about treatment of CML and BCR::ABL1-negative MPN |
| --- | --- | --- |
| 19 | 14 (74%) | 5 (26%) |
| TKI followed by allogeneic SCTx | 5 (36%) 2 | |
| cytoreductive therapy + TKI | 4 (29%) | |
| cytoreductive therapy | 1 (7%) | |
| interferon, followed by asciminib | 1 (7%) | |
| TKI + ruxolitinib | 1 (7%) | |
| no therapy | 2 (14%) | |
Supplemental Figure 3B
| cases MPN + CML | cases with information about treatment of BCR::ABL1-negative MPN and CML | cases without information about treatment of BCR::ABL1-negative MPN and CML |
| --- | --- | --- |
| 55 | 42 (76%) | 13 (24%) |
| TKI followed by allogeneic SCTx | 37 (88%) 1 | |
| TKI + ruxolitinib | 4 (10%) | |
| allogeneic SCTx | 1 (2%) | |
Supplemental Figure 3: Analysis of treatment of CML+MPN and MPN+CML. A, Data on the treatment of patients with CML+MPN were available for 14 cases. 5 patients were treated with tyrosine kinase inhibitor (TKI), 2 of whom received allogeneic stem cell transplantation (SCTx) during follow-up. 4 patients were treated with a combination of TKI and cytoreductive therapy (hydroxyurea, hydroxycarbamide, or anagrelide), 1 received only cytoreductive therapy. 1 patient was treated with interferon followed by asciminib, 1 received a TKI and ruxolitinib; and 2 received no therapy. B, Data on the treatment of patients with MPN+CML were available for 42 cases. 37 patients were treated with tyrosine kinase inhibitor (TKI), 1 of whom received allogeneic stem cell transplantation (SCTx) during follow-up. 4 patients were treated with TKI + ruxolitinib, 1 patients received allogeneic stem cell transplantation after diagnosis of secondary CML.
